# Modulation of Signal Regulatory Protein α (SIRPα) by *Plasmodium* Antigenic Extract: A Preliminary In Vitro Study on Peripheral Blood Mononuclear Cells

**DOI:** 10.3390/microorganisms10050903

**Published:** 2022-04-26

**Authors:** Priscilla da Costa Martins, Hugo Amorim dos Santos de Souza, Carolina Moreira Blanco, Luana Santos-de-Oliveira, Lilian Rose Pratt-Riccio, Cláudio Tadeu Daniel-Ribeiro, Paulo Renato Rivas Totino

**Affiliations:** Laboratório de Pesquisa em Malária, Instituto Oswaldo Cruz, Fundação Oswaldo Cruz, Rio de Janeiro 21040-900, Brazil; priscillamartins0502@gmail.com (P.d.C.M.); hugoamorims@gmail.com (H.A.d.S.d.S.); carolinamoreirablanco@gmail.com (C.M.B.); luana_santos93@yahoo.com.br (L.S.-d.-O.); riccio@ioc.fiocruz.br (L.R.P.-R.); malaria@fiocruz.br (C.T.D.-R.)

**Keywords:** *P. falciparum*, *P. vivax*, LPS, innate immune response, SIRPα

## Abstract

Signal regulatory protein α (SIRPα) is an immunoreceptor expressed in myeloid innate immune cells that signals for inhibition of both phagocytosis and inflammatory response. Malaria parasites have evolutionarily selected multiple mechanisms that allow them to evade host immune defenses, including the modulation of cells belonging to innate immunity. Notwithstanding, little attention has been given to SIRPα in the context of immunosuppressive states induced by malaria. The present study attempted to investigate if malaria parasites are endowed with the capacity of modulating the expression of SIRPα on cells of innate immune system. Human peripheral blood mononuclear cells (PBMC) from healthy individuals were incubated in the presence of lipopolysaccharide (LPS) or crude extracts of *P. falciparum* or *P. vivax* and then, the expression of SIRPα was evaluated by flow cytometry. As expected, LPS showed an inhibitory effect on the expression of SIRPα in the population of monocytes, characterized by cell morphology in flow cytometry analysis, while *Plasmodium* extracts induced a significant positive modulation. Additional phenotyping of cells revealed that the modulatory potential of *Plasmodium* antigens on SIRPα expression was restricted to the population of monocytes (CD14^+^CD11c^+^), as no effect on myeloid dendritic cells (CD14^−^CD11c^+^) was observed. We hypothesize that malaria parasites explore inhibitory signaling of SIRPα to suppress antiparasitic immune responses contributing to the establishment of infection. Nevertheless, further studies are still required to better understand the role of SIRPα modulation in malaria immunity and pathogenesis.

## 1. Introduction

The innate immune system is the first line of defense against pathogens, acting basically through the recognition of pathogen-associated molecular patterns (PAMPs) found in different microorganism classes, including parasitic protozoa [1]. Upon sensing PAMPs by host pattern recognition receptors (PRRs), innate immune cells, such as macrophages, monocytes and dendritic cells (DCs), trigger intracellular signaling pathways leading to antimicrobial and inflammatory responses [2]. This event contributes to the direct elimination of pathogens, as well as the initiation of adaptive immune response mediated by lymphocytes. To ensure survival and propagation, pathogens have evolved a variety of strategies that facilitate their escape from innate immune responses, such as modulation of host cell activation [3,4,5]. Indeed, it has been demonstrated that malaria parasite antigens can inhibit maturation of lipopolysaccharide (LPS)-stimulated DCs both in vitro and in vivo, as well as fail to directly activate DCs in vitro, as shown by the down-regulation of the human leukocyte antigen (HLA-DR) and co-stimulatory molecules, and by reduced cytokine secretion, hampering T-cell responses [6,7,8]. In the same way, impairment of cell functions by malaria parasites has also been reported in monocytes and macrophages, in which suppression of important cellular effector events, such as phagocytosis, oxidative burst, cytokine release and HLA-DR expression, was observed [9,10,11,12,13].

In the context of suppressive immune responses, an immunoreceptor that has gained attention is the Signal Regulatory Protein alpha (SIRPα)—a transmembrane glycoprotein also designated as SHPS-1, CD172a and p84 that is mainly expressed in leukocytes of the myeloid lineage, including monocytes and DCs [14]. SIRPα belongs to the immunoglobulin (Ig) superfamily and presents as a key characteristic an immunoreceptor tyrosine-based inhibition motif (ITIM) in its cytoplasmic tail, which acts negatively in signaling pathways of cell activation [15]. It has been shown that by binding to its ligands, CD47, a ubiquitous cell membrane glycoprotein, and lung surfactant proteins A and D, SIRPα mediates inhibitory signaling of innate immunity functions, preventing, for instance, phagocytosis, proinflammatory cytokine production and DCs maturation [16,17,18,19]. Comprehensibly, it is believed that inhibitory signaling mediated by SIRPα can impact effective innate immune responses against infectious microbes—an issue that has not been widely addressed. To gain insight into SIRPα in malaria, in the present work, we investigate the modulatory effect of *Plasmodium* crude extracts on SIRPα expression in peripheral blood innate immune cells.

## 2. Material and Methods

### 2.1. P. falciparum and P. vivax Antigens

Asexual blood stages of *P. falciparum* (W2 strain) were maintained in continuous in vitro culture according to the method described by Trager and Jensen [20]. Parasites were cultured using O^+^ human red blood cells (RBCs) in RPMI-1640 medium (Sigma, St. Louis, MO, USA) supplemented with 25 mM Hepes (Sigma), 0.2% glucose (Sigma), 23 mM sodium bicarbonate (Sigma), 40 mg/mL gentamycin (Gibco Industries, Big Cabin, OK, USA) and 10% heat inactivated AB^+^ human serum (complete medium). Cultures were maintained at 5% hematocrit at 37 °C under an atmosphere of 5% O_2_, 5% CO_2_ and 90% N_2_ (White Martins Praxair Inc., Rio de Janeiro, Brazil). Parasites were synchronized by repeated sorbitol treatments and parasitized RBCs (pRBCs) were enriched by 60% Percoll density gradient using predominantly mature stage cultures, as described elsewhere [21]. *P. vivax* parasites were obtained from a peripheral blood sample of a patient presenting with non-complicated malaria and the enrichment of mature stages was performed using Percoll 45%, as described by Carvalho et al. [22]. Finally, the pRBCs were suspended in phosphate saline buffer (PBS), sonicated in an ice bath. Protein concentration was then determined using the Qubit Protein Assay Kit (Molecular Probes, Eugene, OR, USA).

### 2.2. PBMC Isolation and Antigenic Stimulation

Heparinized venous blood samples were collected from five clinically healthy individuals, as approved by the Human Research and Ethic Committee of the Oswaldo Cruz Foundation (CAAE 46084015.1.0000.5248). The same individuals were recruited for all experiments and peripheral blood mononuclear cells (PBMCs) were isolated through density gradient centrifugation using Histopaque-1077 (Sigma). Cells were washed twice in RPMI-1640 medium (Sigma) containing 2.05 mM l-glutamine, 25 mM Hepes, and 2.0 g/L sodium bicarbonate and then resuspended in RPMI medium supplemented with 200 U/mL penicillin (Gibco), 200 mg/mL streptomycin (Gibco), and 10% inactivated fetal calf serum (Gibco). Cells (2.5 × 10^5^) were incubated for 24 and 48 h in the absence or presence of *Plasmodium* extracts (0.1–10 μg/mL) or *Escherichia coli* lipopolysaccharides (LPS, 5 μg/mL, Sigma) in 96-well culture plates ( Corning Incorporated, Durham, NC, USA) at 37 °C in 5% CO_2_. PBMCs from the same individuals were used for all experiments.

### 2.3. Flow Cytometry Assay

Expression of SIRPα on PBMC was assayed by flow cytometry using an APC-conjugated anti-SIRPα monoclonal antibody (eBioscience, San Diego, CA, USA). Additionally, PerCP-Cy5.5-conjugated anti-CD11c (BD Pharmingen, San Diego, CA, USA) and PE-conjugated anti-CD14 (eBioscience) antibodies were used to identify monocyte and dendritic cell populations. Briefly, cells (2.5 × 10^5^) were washed in PBS and, subsequently, incubated at 4 °C for 30 min in PBS containing 10% fetal bovine serum (FBS) to block non-specific staining. After incubation, cells were stained with monoclonal antibodies for 40 min at 4 °C in 100 μL PBS containing 1% FBS. Cells were washed twice and finally analyzed by a FACSVerse flow cytometer (Becton Dickinson, Franklin Lakes, NJ, USA). Analysis of flow cytometry data was performed using BD FACSuite software (Becton Dickinson).

### 2.4. Statistical Analysis

Statistical analyses were performed using GraphPad Prism 5.0 software (San Diego, CA, USA) and differences were tested by one-way ANOVA and Tukey’s post-test. A *p*-value of <0.05 was considered statistically significant. Results presented (MFI: mean fluorescence intensity) were normalized to percentage of non-stimulated cells (Control—100%).

## 3. Results and Discussion

It is well known that *Plasmodium* infections suppress lymphoproliferative responses, which negatively impacts the development of an effective antimalarial immunity [23,24,25]. Some immunological mechanisms involved in such immune suppression states have been described and the modulation of innate immune cells by parasites plays a central role [7,26,27]. Thus, in order to study the immunomodulatory potential of malaria parasites on the expression of the innate immune receptor SIRPα, we incubated PBMC from healthy individuals in the presence of crude *P. falciparum* and *P. vivax* extracts, using LPS as a control stimulus.

**Figure 1:**
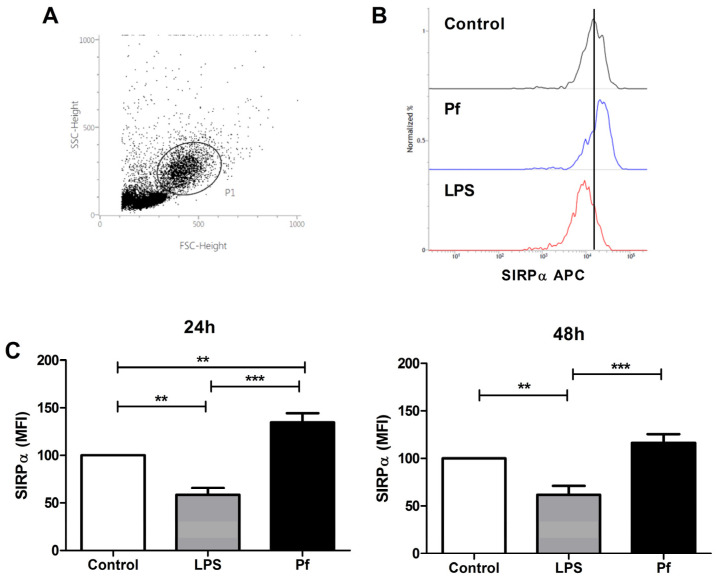
Modulation of SIRPα expression by *P. falciparum* crude extract. PBMCs from healthy individuals were stimulated for 24 and 48 h with *P. falciparum* extract (10 µg/mL) or LPS (5 µg/mL) and SIRPα expression on cell surface of monocyte-like cells was evaluated by flow cytometry using APC-conjugated anti-SIRPα monoclonal antibody. (**A**,**B**) Representative cytometric analysis of SIRPα expression (**B**) in gated monocyte-like cells (**A**; P1) after stimulation with *P. falciparum* antigens (Pf) or LPS. (**C**) Levels of SIRPα expression on monocyte-like cells population (P1), as measured by mean fluorescence intensity (MFI). Non-stimulated PBMCs were used as control (Control). Data are shown as mean ± standard error (SEM) and represent one of two independent experiments performed with PBMC samples from five individuals. **: *p* < 0.01; ***: *p* < 0.001.

As expected, stimulation of PBMCs with LPS by 24 and 48 h resulted in a decreased expression of SIRPα in the population of monocyte-like cells characterized by morphology criteria in flow cytometry analysis (Figure 1). This is in agreement with previous studies in which *Saimiri* monkey PBMCs [28], mouse macrophages [29] and DCs derived from human peripheral blood or mouse bone marrow [30] were stimulated with LPS. Similar downregulation of SIRPα has also been described after viral double-stranded RNA (polyI:C) stimulation of mouse macrophages, in which SIRPα signaling was shown to negatively regulate TLR3-dependent antiviral pathways [31]. Indeed, SIRPα is believed to be a negative modulator of TLR signaling in immune innate cells, as overexpression of SIRPα decreases pro-inflammatory cytokine response, while disruption of SIRPα signaling allows the activation of NF-κβ and an increase in pro-inflammatory cytokine production [29,32]. In this line, it has already been demonstrated, using human monocytic cell line THP-1, that LPS acts as a pro-inflammatory stimulus by triggering SIRPα proteolysis [33].

**Figure 2:**
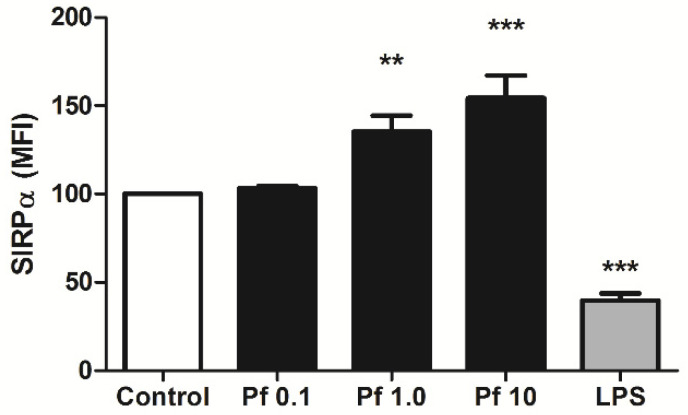
Dose-dependent effect of *P. falciparum* extract on SIRPα expression. PBMCs from healthy individuals were stimulated for 24 h with crescent concentrations of *P. falciparum* extract (Pf; 0.1, 1.0 and 10 µg/mL) or 5 µg/mL LPS and, the levels of SIRPα expression (MFI) on cell surface of monocyte-like cells was evaluated by flow cytometry, as in Figure 1. Data are shown as mean ± standard error (SEM) and represent one of two independent experiments performed with PBMC samples from five individuals. **: *p* < 0.01; ***: *p* < 0.001, as compared with non-stimulated cells (Control).

**Figure 3:**
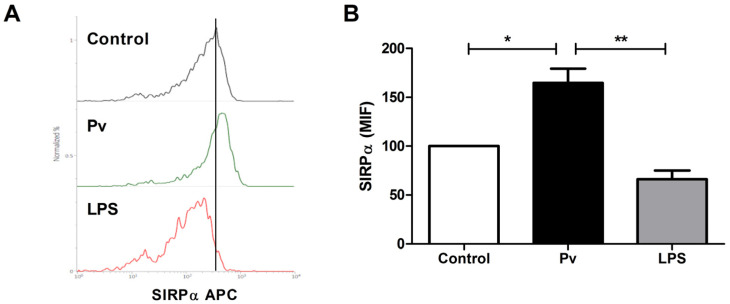
Modulation of SIRPα expression by *P. vivax* crude extract. PBMCs from healthy individuals were stimulated for 24 h with *P. vivax* extract (10 µg/mL) or LPS (5 µg/mL) and SIRPα expression on cell surface of monocyte-like cells was evaluated by flow cytometry using APC-conjugated anti-SIRPα monoclonal antibody. (**A**) Representative histogram analysis of SIRPα expression in gated monocyte-like cells after stimulation with *P. vivax* antigens or LPS. (**B**) Levels of SIRPα expression on monocyte-like cells population, as measured by mean fluorescence intensity (MFI). Non-stimulated PBMCs were used as control (Control). Data are shown as mean ± standard error (SEM) and represent one of two independent experiments performed with PBMC samples from five individuals. *: *p* < 0.05; **: *p* < 0.01.

In marked contrast to LPS, *P. falciparum* crude extract modulated positively SIRPα expression in monocyte-like cells after 24 h incubation (Figure 1), an effect that was shown to be dose-dependent (Figure 2). Similar results were observed when *P. vivax* antigens were used to stimulate PBMC (Figure 3). Thus, although *P. vivax* and *P. falciparum* can cause different clinical and immunological outcomes during acute infection [34,35], the early induction of SIRPα expression might be a common feature avoiding initial parasite clearance and, consequently, allowing the establishment of blood stage. Indeed, besides promoting the engulfment of tumor cells by macrophages in vitro and in vivo [36], disruption of SIRPα signaling can attenuate parasite burden in the course of *P. berghei* ANKA infection in mice, as well as enhance in vitro phagocytosis of *P. falciparum*-pRBCs, which implicates SIRPα as an important component controlling malaria blood-stage [37]. Moreover, such involvement of SIRPα in the control of pathogen growth was also demonstrated in two other models, i.e., pneumonia and melioidosis. In the first model, the impaired phagocytic activity of alveolar macrophages against *Escherichia coli* and *Staphylococcus aureus*, imposed by primary pneumonia, was related to augmented SIRPα expression, which increased the susceptibility of mice to secondary pneumonia [38]. In contrast, the intracellular killing of the causative agent of melioidosis, *Burkholderia pseudomallei*, was associated with deflagration of TLR signaling and subsequent inducible nitric oxide synthase response, triggered as a result of downregulation of SIRPα expression in infected macrophages [32]. Nevertheless, both the stimuli and pathways regulating SIRPα expression remain largely unknown.

**Figure 4:**
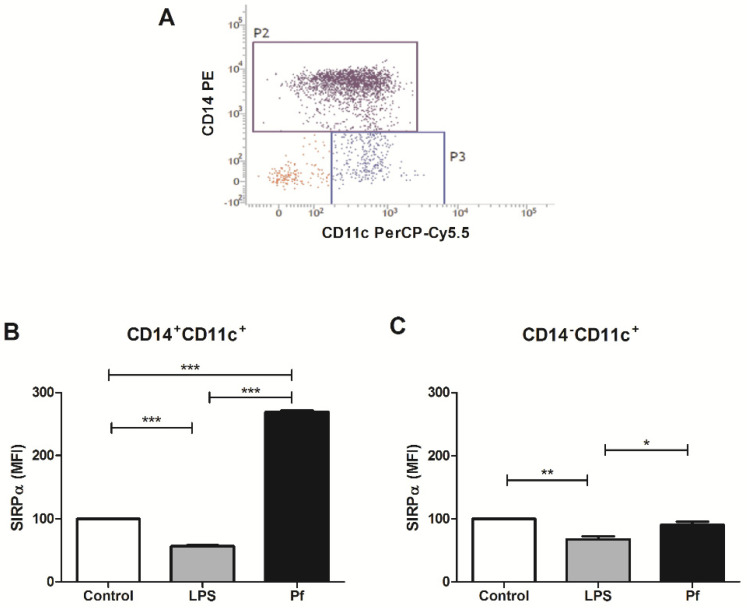
Cell type-specific modulation of SIRPα expression by *P. falciparum* crude extract. PBMCs from healthy individuals were stimulated for 24 h with *P. falciparum* extract (10 µg/mL) or LPS (5 µg/mL) and SIRPα expression on cell surface of monocytes and dencritic cells was evaluated by flow cytometry. (**A**) Representative phenotyping analysis of classical monocytes (P2; CD14^+^CD11^+^) and myeloid dendritic cells (P3; CD14^−^CD11^+^) populations. (**B**,**C**) Levels of SIRPα expression in gated monocytes (**B**) and dendritic cells (**C**), as measured by mean fluorescence intensity (MFI). Non-stimulated PBMCs were used as control (Control). Data are shown as mean ± standard error (SEM) and represent one of two independent experiments performed with PBMC samples from five individuals. *: *p* < 0.05; **: *p* < 0.01; ***: *p* < 0.001.

We finally investigated if *Plasmodium* antigens differentially modulate the innate immune cells by performing basic phenotyping for classical monocytes (CD14^+^CD11c^+^) and myeloid dendritic cells (CD14^−^CD11c^+^), as classified previously [39]. As shown in Figure 4, *P. falciparum* extract positively modulated the expression of SIRPα in monocytes, but had no effect on dendritic cells population, whose susceptibility to negative modulation by LPS was similar to monocytes (Figure 4). It is not surprising, however, that subsets of innate immune cells, upon stimulation with certain pathogens, can respond through distinct signaling pathways, thus contributing in opposite ways to the immune response against the infection [40,41,42,43]. Under this premise, it is possible that the initial and transient upregulation of SIRPα induced by malaria parasites solely in monocytes (Figure 1) could favor the establishment of blood infection at the very early phase, in which the parasite load is still limited, while preserving the role of dendritic cells in priming T cell response. Consistent with this possibility, it was already demonstrated that the activity of dendritic cells and macrophages suffers temporal alterations in the course of experimental malaria [44,45] and that, for instance, the impairment of dendritic cells to stimulate T cells was restricted to late, but not early, phases of blood-stage infection with *P. chabaudi* or *P. yoelii* [46,47].

In conclusion, our work shows that *Plasmodium* crude extracts can positively stimulate the expression of SIRPα in cells of the innate immune system, representing a putative mechanism of parasite evasion contributing to the early establishment of blood-stage infection. Nevertheless, further studies, exploring the dynamic of SIRPα expression in the course of infection and its relation to both immune responses and parasite elimination, are still required for a better understanding of the role of SIRPα in malaria. In this regard, a study with malaria patients from the Brazilian Amazon, where *P. falciparum* and *P. vivax* are endemic, is currently in progress.

## Data Availability

The data of the study are contained within the article.
